# Smoking

**Published:** 1865-11

**Authors:** 


Smoking.—A new "warning to smokers" is found in an analysis of the results of the contents of one good Havana cigar, which yields when its smoke is condensed, a sufficient amount of poisonous matter to induce active convulsions in a rabbit. Six pipes of common shag tobacco will yield sufficient poison to destroy a rabbit in three minutes.

